# Diagnostic Utility of Endoscopic Features and Endoscopic Ultrasonography for Ulcerative Colitis-Associated Neoplasia: A Retrospective Study on the Role of Endoscopic Submucosal Dissection as a Total Biopsy

**DOI:** 10.3390/cancers18091492

**Published:** 2026-05-06

**Authors:** Saki Yoshida, Yoriaki Komeda, Masashi Kono, Kohei Handa, Tomoyuki Nagai, Satoru Hagiwara, Shunsuke Omoto, Mamoru Takenaka, Hiroshi Kashida, George Tribonias, Koji Daito, Junichiro Kawamura, Masatoshi Kudo

**Affiliations:** 1 Department of Gastroenterology and Hepatology, Faculty of Medicine, Kindai University, Osaka 590-0197, Japan; sakiyyyy@gmail.com (S.Y.); kono@med.kindai.ac.jp (M.K.); g21082@edu.med.kindai.ac.jp (K.H.); tomoyukinagai@mac.com (T.N.); hagi-318@hotmail.co.jp (S.H.); shunsuke.oomoto@gmail.com (S.O.); mamoxyo45@gmail.com (M.T.); kashi-md@xf6.so-net.ne.jp (H.K.); m-kudo@med.kindai.ac.jp (M.K.); 2 Department of Gastroenterology, Red Cross Hospital, 115 26 Athens, Greece; g.tribonias@gmail.com; 3 Department of Surgery, Faculty of Medicine, Kindai University, Osaka 590-0197, Japan; daito@med.kindai.ac.jp (K.D.); kawamuraj@med.kindai.ac.jp (J.K.)

**Keywords:** endoscopic submucosal dissection, endoscopic ultrasound, sporadic neoplasia, ulcerative colitis, ulcerative colitis-associated neoplasia

## Abstract

Background/Objectives: Ulcerative colitis (UC)-associated neoplasia (UCAN) often presents as flat lesions with indistinct margins, and biopsy sensitivity is limited. Therefore, we evaluated endoscopic criteria to distinguish UCAN from sporadic neoplasia, assessing the accuracy of endoscopic ultrasonography (EUS) for invasion depth and the role of endoscopic submucosal dissection (ESD) as a “total biopsy.” Methods: We reviewed 212 endoscopically treated neoplastic lesions in UC-affected mucosa (April 2016–January 2025). We compared preoperative diagnoses using macroscopic type, pit pattern, and the Japan Narrow-Band Imaging Expert Team classification with final histology. We compared depth estimates with pathology in 10 UCAN-suspected lesions undergoing EUS. Lesions < 2 cm underwent conventional endoscopic resection, whereas those ≥ 2 cm underwent ESD. Results: No UCAN was found in 189 lesions < 2 cm. Among 23 ESD lesions, 8 suspected sporadic lesions were non-UCAN. Of 15 UCAN-suspected lesions, 8 were UCAN, 6 sporadic, and 1 inflammatory. For positive T1b or deeper invasion, the sensitivity, specificity, positive and negative predictive values, and overall accuracy of EUS were 50.0%, 100%, 100%, 88.9%, and 90.0%, respectively. EUS depth assessment agreed with pathology in 9/10 cases; nine lesions were T1a or shallower, and one was T1b. Third-layer thickening occurred in two lesions—both UCAN—including the T1b cancer. In ESD cases, redness and a VI pit pattern were independent predictors of UCAN. Conclusions: EUS-based depth assessment is useful for determining optimal treatment strategies. Beyond therapy, ESD enables comprehensive histologic assessment of the entire lesion, functioning as a total biopsy to guide management while preserving bowel function.

## Simple Summary

Patients with long-standing ulcerative colitis are at an increased risk of developing cancer in inflamed areas of the colon. Early detection of these cancers is difficult, as they often appear flat and blend with the surrounding tissue. Moreover, standard biopsy techniques may miss cancerous areas, and traditional endoscopic imaging can be unreliable. In this study, we evaluated advanced endoscopic techniques, including magnifying imaging and ultrasound, to determine how accurately they can identify these cancers and how deeply the cancers have invaded the colon wall. We also examined the use of a specialized procedure, endoscopic submucosal dissection, which enables physicians to remove the entire lesion for both treatment and diagnosis. Our findings suggest that combining these techniques improves cancer detection and staging, which may help doctors make better treatment decisions and preserve bowel function in affected patients.

## 1. Introduction

Recently, the rising prevalence of ulcerative colitis (UC) combined with advances in medical therapy has resulted in a growing number of patients living with this long-standing disease. Notably, the risk of developing UC-associated neoplasia (UCAN) increases with disease duration, reaching 1.6%, 8.3%, and 18.4% at 10, 20, and 30 years, respectively [1]. Although total proctocolectomy is the standard treatment for UCAN, the feasibility of endoscopic resection has been increasingly explored [2]. However, because UCAN arises from a background of chronic inflammation, lesion borders are often indistinct, and lesions commonly present as flat or non-polypoid lesions. Consequently, distinguishing UCAN from sporadic neoplasia is challenging. Moreover, repeated inflammation leads to fibrosis and mucosal alterations, further complicating the accurate assessment of lesion margins and invasion depth.

According to the 2024 Japanese Guidelines for Inflammatory Bowel Disease-Associated Gastrointestinal Neoplasia, the indications for endoscopic submucosal dissection (ESD) are limited to lesions no more advanced than low-grade dysplasia (LGD) that meet the following three criteria: (1) clearly demarcated lesions allowing accurate assessment of the horizontal extent; (2) absence of moderate-to-severe inflammation surrounding the lesion; and (3) high likelihood of *en bloc* resection with negative vertical and horizontal margins [3]. As indicated, diagnostic ESD for suspected UCAN is performed when these conditions are satisfied, and the lesion is expected to correspond to high-grade dysplasia (HGD), Tis, or T1a. When the ESD specimen is diagnosed as LGD, total proctocolectomy is not indicated, and strict endoscopic surveillance is recommended. In cases where cancer or HGD is confirmed, and the lesion is considered to arise in the background of chronic inflammation, total proctocolectomy is recommended according to the Evidence-based Clinical Practice Guidelines for Colonic Polyp 2020 (2nd Edition) [4]. However, the sensitivity of preoperative biopsy-based diagnosis for UCAN is as low as 72.2%, and discrepancies between preoperative biopsy findings and final pathological diagnoses are common [5]. Therefore, improving preoperative diagnostic accuracy remains an urgent clinical issue. In this context, ESD may provide diagnostic value both as a therapeutic modality and a “total biopsy,” enabling comprehensive pathological evaluation of the entire lesion.

Pouchitis develops in 50–79% of patients post-total proctocolectomy with ileal pouch formation, leading to increased stool frequency, fecal incontinence, and significant postoperative quality-of-life impairment [6]. Thus, establishing an accurate preoperative diagnosis is critical for selecting an optimal treatment strategy that balances oncological safety with the preservation of bowel function. Among these patients, a subset develops chronic or antibiotic-refractory pouchitis, for which optimal management strategies remain incompletely established. Moreover, endoscopic ultrasonography (EUS) is a useful modality for evaluating layer structure and invasion depth; however, few studies have assessed its utility and diagnostic accuracy, specifically for UCAN [7].

Given these clinical challenges, in the present study, we retrospectively investigated endoscopically treated neoplastic lesions arising in UC-affected mucosa at our institution, focusing on: (1) the diagnostic ability of endoscopic findings to distinguish UCAN from non-UCAN lesions (sporadic adenoma, sessile serrated lesions [SSLs], and hyperplastic/inflammatory polyps) and (2) EUS accuracy in assessing invasion depth, aiming to improve diagnostic precision for UCAN.

## 2. Materials and Methods

### 2.1. Study Population

In this retrospective study, we included 181 patients with 212 lesions arising in UC-affected mucosa who underwent endoscopic treatment at Kindai University Hospital between April 2016 and January 2025. Two main analyses were performed in this study.

### 2.2. Diagnostic Histopathologic Criteria

Histopathological differentiation between UCAN and sporadic adenoma was performed based on established morphological criteria, evaluation of the surrounding mucosa, and ancillary immunohistochemical findings (e.g., p53 and Ki-67). Specifically, lesions arising in chronically inflamed mucosa with architectural distortion and dysplasia extending to the adjacent mucosa were considered UCAN. In contrast, polypoid lesions with adenomatous architecture arising in non-inflamed mucosa were classified as sporadic adenomas.

The diagnostic assessment was performed in accordance with the Riddell dysplasia classification [8], the World Health Organization classification of digestive system tumors [9], and current staging systems [10,11].

### 2.3. Diagnostic Evaluation Based on Endoscopic Findings

Preoperative diagnoses were made using macroscopic classification, pit pattern analysis [12], and the Japan Narrow-Band Imaging (NBI) Expert Team (JNET) classification [13]. These findings were compared with the final pathological diagnoses to evaluate the diagnostic accuracy in distinguishing UCAN from sporadic neoplasia.

### 2.4. Endoscopic Treatment Strategy

**Figure 1:**
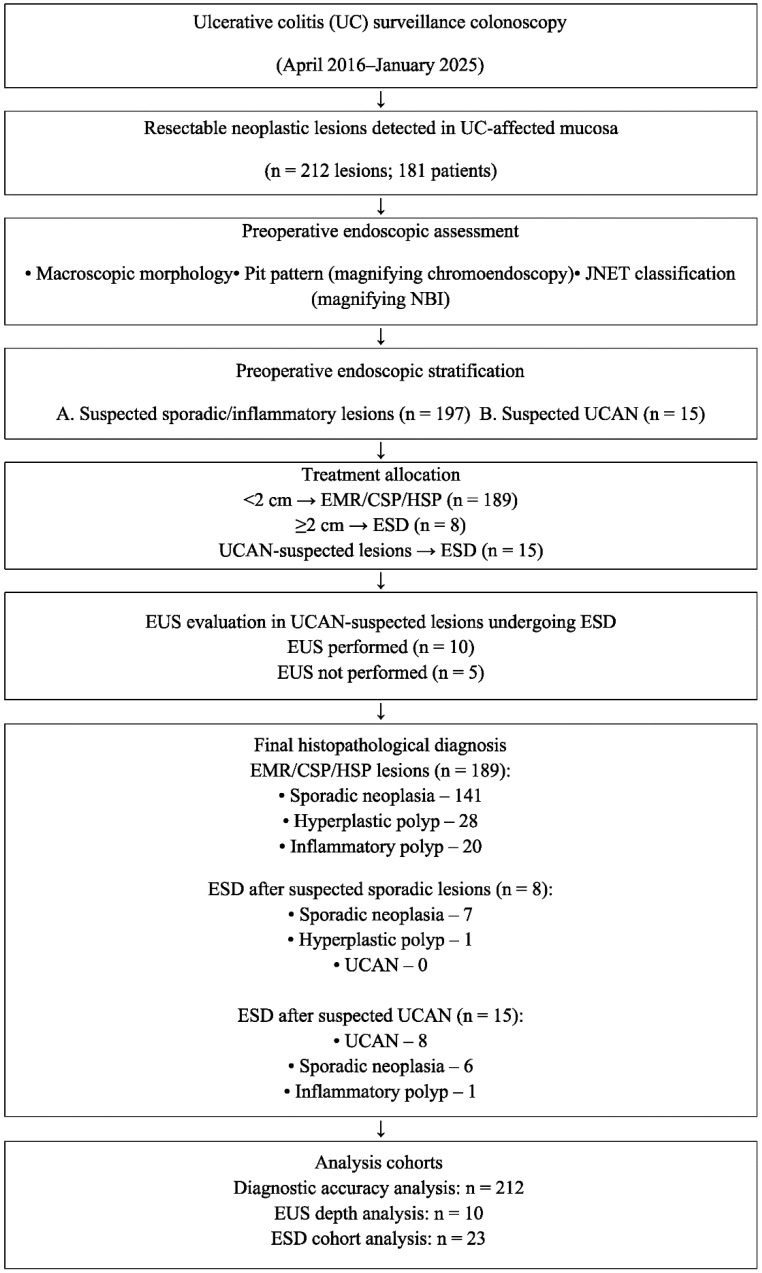
STROBE flow diagram of the lesion detection and treatment strategy. CSP, cold snare polypectomy; EMR, endoscopic mucosal resection; ESD, endoscopic submucosal dissection; EUS, endoscopic ultrasonography; HSP, hot snare polypectomy; JNET, Japan Narrow-Band Imaging Expert Team; NBI, narrow-band imaging; UCAN, ulcerative colitis-associated neoplasia.

At our institution, lesions < 2 cm in diameter are treated using endoscopic mucosal resection (EMR), cold snare polypectomy (CSP), or hot snare polypectomy (HSP), whereas ESD is performed for lesions ≥ 2 cm (Figure 1). Selection of the treatment modality (ESD vs. EMR/CSP/polypectomy) was determined based on both lesion size and magnifying endoscopic findings.

Magnifying endoscopic diagnosis was performed using the JNET [13] and pit pattern classifications (indigo carmine and crystal violet staining) [12], which are commonly employed for the evaluation of sporadic colorectal neoplasia. Observations were conducted at 80–100× magnification.

For lesions < 2 cm exhibiting findings typical of sporadic neoplasia or inflammatory lesions, EMR, CSP, or polypectomy was selected. No UCAN cases were included in this treatment group. Lesions that did not meet these diagnostic criteria and were suspected of UCAN were treated with ESD, even if they were smaller than 2 cm.

In this study, ESD was indicated when the lesion is expected to correspond to HGD, Tis, or T1a, and the following criteria are satisfied: (1) clear, demarcated lesions allowing accurate assessment of horizontal extent; (2) absence of moderate-to-severe inflammation surrounding the lesion that could compromise margin evaluation; and (3) high likelihood of achieving *en bloc* resection with negative vertical and horizontal margins based on endoscopic assessment.

All cases in this study were in endoscopic mucosal remission (up to Mayo Endoscopic Subscore [MES] 1). Four-quadrant biopsies were collected in a stepwise fashion around the lesion to confirm the absence of dysplasia in the surrounding mucosa, and p53 immunohistochemical staining was performed as previously described [14]. ESD was performed only when dysplasia was not detected and used as an adjunctive modality when UCAN was suspected.

### 2.5. Accuracy of EUS for Depth Assessment

**Figure 2:**
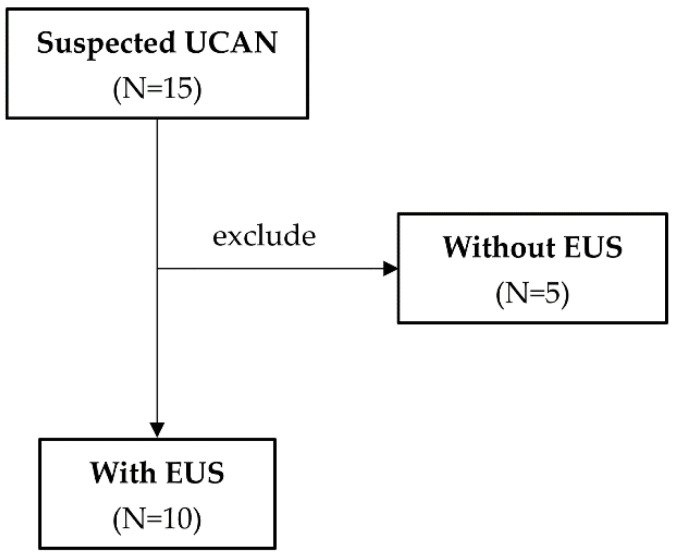
Cases in which ulcerative colitis-associated neoplasia (UCAN) was suspected and endoscopic ultrasonography (EUS) was performed.

Among the lesions preoperatively suspected to be UCAN and treated with ESD, 10 underwent EUS. EUS-assessed invasion depth was compared with the final pathological depth to evaluate diagnostic accuracy (Figure 2). For lesions suspected of UCAN, EUS was performed in cases scheduled for ESD, enabling the evaluation of the full-layer wall structure.

In this study, EUS assessment for UCAN focused on changes in the intestinal wall layer structure. Normally, the five-layer wall structure is clearly visualized; however, the following findings were defined as abnormal: blurring of the layered structure, layer disruption, and loss of the layered structure, particularly loss of continuity of the third layer (submucosa) and hypoechoic findings suggestive of invasion into the fourth layer (muscularis propria).

Overall, abnormal EUS findings were defined as disruption or irregularity of the wall layer structure, particularly hypoechoic changes suggestive of invasion from the submucosa to the muscularis propria.

### 2.6. Statistical Analysis

Continuous variables are expressed as mean ± standard deviation, and categorical variables are presented as numbers and percentages. The normality of continuous variables was assessed using the Shapiro–Wilk test. Depending on the distribution, comparisons between groups were performed using Student’s *t*-test or Mann–Whitney U test, whereas categorical variables were analyzed using the chi-square or Fisher’s exact test, as appropriate. Logistic regression analysis was performed to explore factors associated with UCAN in lesions treated with ESD. Odds ratios (ORs) with 95% confidence intervals (CIs) were reported, and statistical significance was defined as *p* < 0.05.

The diagnostic accuracy for identifying mucosal or superficial submucosal carcinoma was calculated using sensitivity, specificity, positive predictive value (PPV), and negative predictive value (NPV), together with 95% CIs. All statistical analyses were performed using JMP software (v.13; SAS Institute, Cary, NC, USA) and IBM SPSS (v.27; IBM Corp., Armonk, NY, USA).

In patients who underwent ESD, logistic regression analysis was performed to identify factors associated with UCAN compared with those for non-UCAN lesions, including SSLs, tubular adenomas, and inflammatory polyps. The variables examined included sex, disease duration, UC phenotype, MES, lesion location, lesion size, macroscopic type, color tone, pit pattern on chromoendoscopy, JNET classification on NBI, and Takabayashi classification.

This study was approved by our institutional ethics committee (Approval No.: 28-093).

## 3. Results

### 3.1. Pathological Outcomes of Lesions

Of the 212 endoscopically treated lesions, 189 lesions < 2 cm were treated with EMR, CSP, or polypectomy, whereas 23 lesions ≥ 2 cm were treated with ESD (Table 1). Among the lesions treated with EMR, CSP, or polypectomy, the final pathological diagnoses included 141 cases of sporadic neoplasia, 28 hyperplastic polyps, and 20 inflammatory polyps. No cases of UCAN were identified in lesions measuring <2 cm.

Among these ESD cases, eight lesions were preoperatively suspected to be sporadic neoplasia, and 15 were suspected to be UCAN (Table 1 and Table 2). Of the eight lesions preoperatively suspected to be sporadic neoplasia and treated with ESD, final pathology confirmed seven cases of sporadic neoplasia and one hyperplastic polyp, with no cases of UCAN observed. Conversely, among the 15 lesions preoperatively suspected to be UCAN, the final pathology confirmed 8 UCAN, 6 sporadic neoplasia, and 1 inflammatory polyp case. Among the 23 lesions treated with ESD, the clinicopathological features and clinical outcomes of each case are summarized in Table 3.

**Figure 3:**
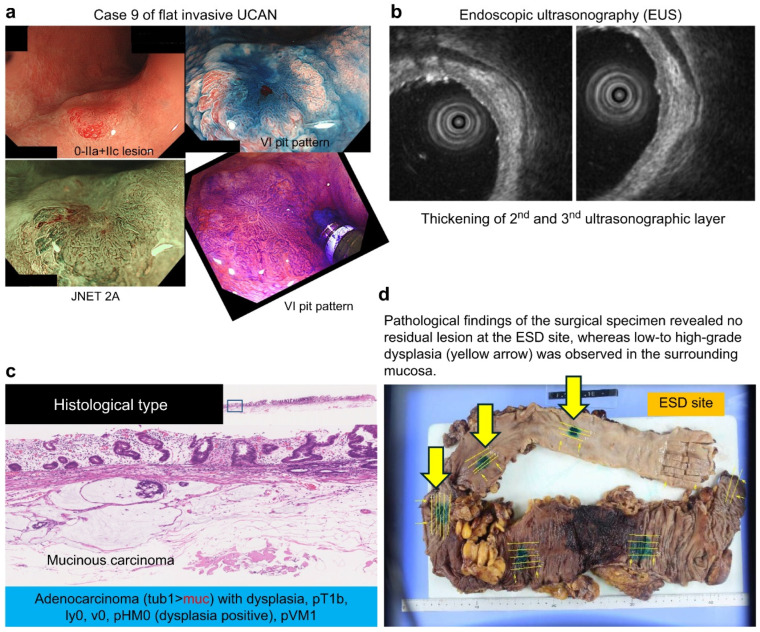
Flat invasive UCAN (Case 9). (**a**) Endoscopic findings of a 0-IIa+IIc lesion with a type VI pit pattern and JNET type 2A. (**b**) EUS showing thickening of the second and third layers. (**c**) Histology revealing adenocarcinoma with dysplasia (pT1b). (**d**) Surgical specimen showing no residual tumor at the ESD site and surrounding dysplasia.

**Figure 4:**
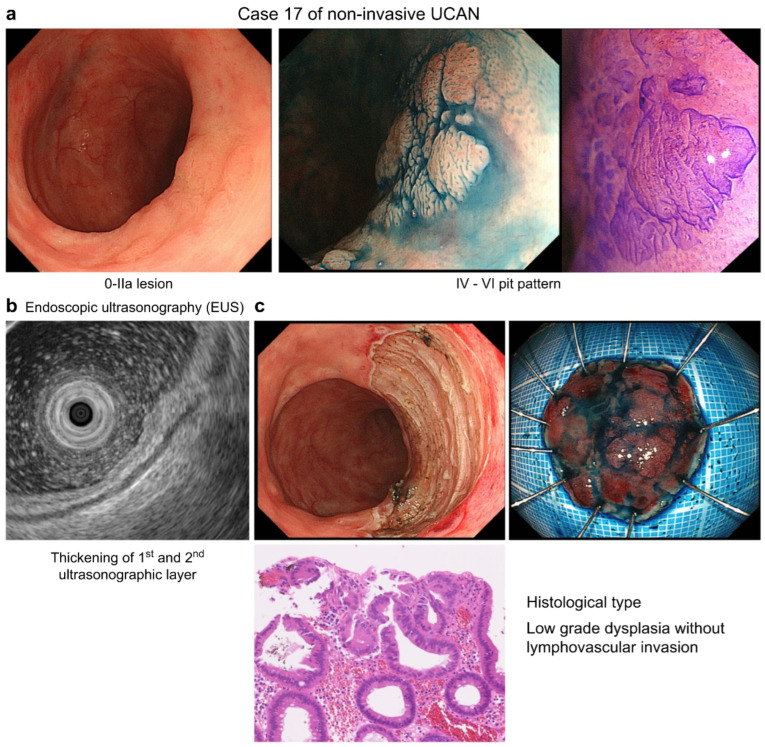
Non-invasive UCAN (Case 17). (**a**) Endoscopic findings of a 0-IIa lesion with a mixed IV–VI pit pattern. (**b**) EUS showing thickening of the first and second layers. (**c**) Histology demonstrating low-grade dysplasia.

**Figure 5:**
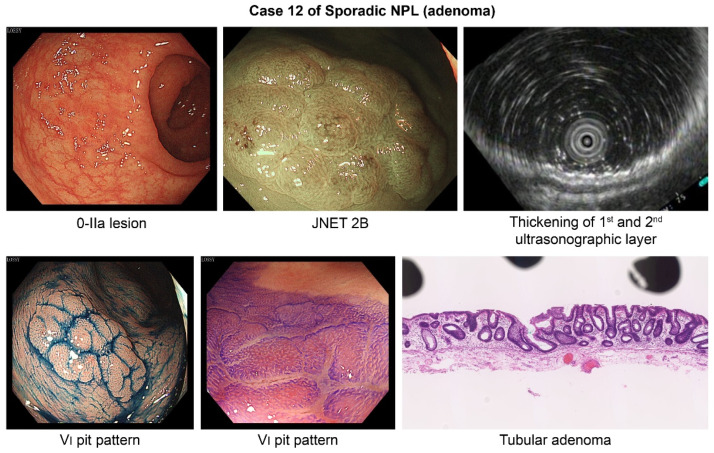
Sporadic non-polypoid lesion (adenoma) (Case 12). Endoscopic findings of a 0-IIa lesion with JNET type 2B and a type VI pit pattern. EUS showing thickening of the first and second layers. Histology confirming tubular adenoma.

Representative examples of flat-invasive UCAN, non-invasive UCAN, and sporadic non-polypoid lesions (adenoma) are presented in Figure 3, Figure 4 and Figure 5, respectively.

### 3.2. Diagnostic Performance of Preoperative Endoscopic Evaluation

When all endoscopically treated lesions were preoperatively categorized as “UCAN versus non-UCAN (including sporadic adenoma)” and compared with the final pathology (“UCAN versus non-UCAN”), the diagnostic sensitivity, specificity, PPV, NPV, and overall accuracy were 100%, 96.6%, 53.3%, 100%, and 96.7%, respectively.

### 3.3. EUS Depth Assessment

When T1b or deeper invasion was defined as a positive finding, the sensitivity, specificity, PPV, NPV, and overall accuracy of EUS were 50.0% (95% CI: 1.3–98.7%), 100% (95% CI: 63.1–100%), 100% (95% CI: 2.5–100%), 88.9% (95% CI: 51.8–99.7%), and 90.0% (95% CI: 55.5–99.7%), respectively, in Table 3.

After excluding 5 lesions suspected to be UCAN for which EUS was not performed, 10 cases were included in the EUS depth analysis. The median patient age and disease duration were 60 and 27 years, respectively. Pit pattern classifications varied (types II, IIIs, and IV, and mildly type VI), whereas the JNET classification confirmed two lesions as type 2B and the remaining ones as type 2A. EUS findings revealed that eight lesions were confined to the second layer without third-layer thickening or narrowing, whereas two lesions exhibited third-layer thickening, raising the suspicion of T1b or deeper invasion.

The final pathological diagnoses included three sporadic neoplasia, six UCAN, and one inflammatory polyp case. Both lesions with third-layer thickening were UCAN: one was T1b carcinoma (Case 9), and the other was Tis (Case 10).

When the 10 EUS-evaluated lesions were classified as “T1a or shallower versus T1b or deeper” and compared with the final pathological depth, EUS assessment was concordant in all but one case (Case 9), resulting in a diagnostic accuracy of 90%.

In the present series, two cases exhibited thickening of the third layer, and both were suspected to be T1b on EUS. In one case, the final diagnosis was T1b mucinous carcinoma, which was consistent with the EUS findings (presented case). In the other case, the final diagnosis was Tis (intramucosal lesion); however, the apparent thickening was likely due to lymphoid follicles. Therefore, only one case was discordant.

### 3.4. Factors Associated with Ulcerative Colitis-Associated Neoplasia in ESD Cases

Logistic regression analysis was performed to compare UCAN lesions with non-UCAN lesions (including SSL, tubular adenoma, and inflammatory polyps) for the following variables: sex, disease duration, disease phenotype, MES, lesion location, lesion size, macroscopic type, color tone, pit pattern, JNET classification, and Takabayashi classification.

Among these variables, reddened appearance (color tone; *p* = 0.049) and pit pattern findings on chromoendoscopy (*p* = 0.049) emerged as the only significant independent predictors of UCAN (Table 4).

### 3.5. Final Outcomes of Diagnostic ESD and Adverse Events

The overall *en bloc* and curative resection rates were 91.3% and 95.7%, but 100% and 87.5% among UCAN lesions, respectively. The mean ESD procedure time was 116 min overall and 138 min in UCAN cases. The mean length of hospital stay was 6.7 days overall and 6.6 days in UCAN cases.

Regarding adverse events, one case of delayed perforation occurred in the overall cohort, which was observed only in sporadic neoplasia. Based on the final pathological diagnosis, two UCAN cases with cancer and two with HGD underwent total proctocolectomy. Among these four surgical cases, metachronous lesions were identified in three cases (75%) on pathological examination.

### 3.6. Presentation of Ulcerative Colitis-Associated Neoplasia Prevalence

The proportion of UCAN in this study can be presented as follows:

(1) Proportion of UCAN among all endoscopically treated lesions (n = 212): among the 212 endoscopically treated lesions in UC-associated mucosa, eight were finally diagnosed as UCAN on pathological examination. Thus, the proportion of UCAN was 8/212 (3.77%, approximately 3.8%).
(2) Proportion of UCAN among ESD-treated lesions (n = 23): among the 23 lesions treated with ESD, eight were diagnosed as UCAN. Thus, the proportion of UCAN among ESD-treated lesions was 8/23 (34.78%, approximately 34.8%).
(3) Proportion of final UCAN diagnoses among lesions suspected of UCAN and treated with ESD (n = 15): among the 15 lesions that were preoperatively suspected of UCAN and treated with ESD, eight were finally diagnosed as UCAN. Thus, the proportion was 8/15 (53.33%, approximately 53.3%), corresponding to the PPV reported in the study findings.

### 3.7. Presentation of Likelihood Ratios (LR+ and LR−)

The LRs can be interpreted as follows:

LR+ = ∞, which indicates that when EUS suggests T1b invasion, the diagnosis is highly reliable, demonstrating a strong rule-in value.

LR− = 0.5, which indicates that even when EUS suggests T1a invasion, the ability to exclude deep invasion remains limited, reflecting a relatively weak rule-out value.

In summary, EUS appears to be useful for confirming deep invasion but less reliable for excluding it.

## 4. Discussion

UCAN arises in the context of chronic inflammation, fibrosis, and regenerative epithelia. Consequently, unlike sporadic neoplasia, UCAN often exhibits indistinct margins and a flat or non-polypoid morphology on endoscopy. Pit pattern classification, originally developed for non-inflamed colorectal mucosa, is not directly applicable to UC-affected mucosa, where inflammation and regenerative changes frequently alter surface structure. Therefore, UCAN diagnosis based solely on surface microstructure has inherent limitations, and improving preoperative assessment accuracy remains a major clinical challenge.

This limitation is consistent with a recent systematic review by Maeda et al., which demonstrated that even advanced endoscopic technologies have only a limited capability for the optical characterization of neoplasia in UC, as chronic inflammation and bottom-up growth frequently obscure conventional surface patterns [15]. Regarding white-light endoscopy, Hisabe et al. reported a retrospective analysis of 27 lesions in which white-light observation was performed within two years before detection, and the same lesion site could be identified. Among pTis and pT1 lesions (n = 11), 81.8% were endoscopically invisible, whereas 68.8% of pT2 and pT3 lesions (n = 16) were also undetectable, highlighting the limited sensitivity of white-light imaging for UCAN detection [16].

In the present study, pit pattern- and NBI-based diagnoses exhibited a high sensitivity (100%) and specificity (96.6%) for UCAN; however, the PPV remained low (53.3%). This indicates that although no UCAN lesions were missed, a substantial proportion of lesions were overdiagnosed as UCAN, likely owing to inflammation-related or regenerative alterations that mimic neoplastic features. Takabayashi et al. recently re-evaluated pit and vascular pattern classifications using magnifying endoscopy and identified several UCAN-characteristic features, including small, round, villous, irregular, and mesh patterns [17]. Pit patterns reflect the epithelial surface structure, whereas vascular patterns reflect microvascular architecture within the lamina propria. Combining these two patterns may enhance diagnostic accuracy. Specifically, the mesh pattern reflects inflammation-induced angiogenesis, which is characterized by a reticular vascular network. Takabayashi et al. confirmed that these characteristic findings can highlight the structural heterogeneity that is not adequately captured using conventional pit pattern criteria [17]. Notably, when these criteria were applied to our dataset, the PPV improved to 80%; however, 20% of the lesions were still non-UCAN, underscoring persistent diagnostic limitations.

Shinagawa et al. reported additional endoscopic UCAN features, including pit destruction, irregular crypt enlargement, vascular irregularity, and a unique “pinecone pattern” [18]. This pinecone-like appearance reflects fused, hypertrophic pits resulting from a mixture of regenerative epithelium and neoplastic elements and is considered relatively specific for UCAN. Histologically, this pattern corresponds to fused dysplastic glands and epithelial bilayering, representing transitional areas between regeneration and neoplasia [18]. Moreover, Kawasaki et al. demonstrated that although highly irregular VI and VN pit patterns can suggest deep submucosal invasion with high specificity, their sensitivity remains limited (25–50%) [19]. In UC, where submucosal fibrosis is present beneath the lesion, pit pattern and NBI findings alone may either underestimate or overestimate the invasion depth. In addition, Suzuki et al. observed submucosal fibrosis in nearly all cases of colitis-related dysplasia treated by ESD, highlighting the technical difficulty of endoscopic resection in UC-associated lesions and the limitations of preoperative assessment based solely on surface findings [20]. This challenge is clinically important because severe submucosal fibrosis is a major technical factor during ESD for UCAN, further supporting the need for a more integrated preoperative assessment beyond surface-based optical diagnosis alone [21,22]. Therefore, in our study, we combined magnifying endoscopy with EUS to overcome the limitations of surface-based assessments that are distorted by inflammatory and fibrotic changes.

Nishio et al. reported that magnifying NBI yielded a sensitivity of 66.5%, a specificity of 79.0%, and an overall accuracy of 71.8% for diagnosing UCAN, with a higher specificity among expert endoscopists than that among trainees (83% vs. 70%). Moreover, polypoid lesions exhibited a higher sensitivity (92%) but lower specificity (61%), whereas non-polypoid lesions exhibited the opposite trend. The inter-observer agreement was only moderate (κ = 0.411), and in cases in which all observers misdiagnosed UCAN, the vascular and surface patterns were misinterpreted as non-neoplastic. These findings suggest that although magnifying NBI provides some diagnostic utility, its sensitivity for non-polypoid UCAN is limited, highlighting the need for standardized diagnostic criteria that incorporate UC-specific features [22]. In the present study, EUS yielded 90% concordance with the pathological depth of invasion, supporting its utility in preoperative decision-making. In a small, selected subset of lesions suspected of UCAN in which EUS was performed, EUS appeared to provide supplementary information for pre-treatment assessment. As only one lesion exhibited pathological submucosal invasion (T1b), the present analysis should be interpreted as exploratory rather than confirmatory.

Notably, among the two lesions with third-layer thickening on EUS, one was confirmed as T1b carcinoma and could have been appropriately triaged for surgical management preoperatively. In another case, deep submucosal invasion was associated with mucinous carcinoma. Although surgical resection was recommended, the patient initially opted for ESD. Another important consideration is the emerging role of diagnostic ESD as a “total biopsy” for UCAN. In Japanese gastroenterology, endoscopic resection provides clinical value both as a treatment modality and a means of obtaining a complete excisional specimen for accurate histologic assessment and subsequent management planning [14,23,24]. Kinoshita et al. reported that the sensitivity of UCAN biopsy is only 72.2%, with approximately 30% discordance between preoperative biopsies and final pathological diagnoses [5]. As UCAN often includes inflammatory and regenerative components, partial biopsies may inadequately sample dysplastic tissue, thereby complicating definitive diagnosis.

Recent reviews have emphasized that the management of visible dysplasia in inflammatory bowel disease (IBD) requires an integrated strategy encompassing lesion detection, optical characterization, endoscopic resection, and post-resection surveillance [25]. Consistent with this concept, recent multicenter studies support the role of endoscopic resection for visible colorectal neoplasia in patients with IBD [24]. Notably, a US multicenter study conducted by Ngamruengphong et al. demonstrated that ESD is feasible and effective for colorectal dysplasia in IBD. These findings were reinforced by the nationwide GETAID/SFED cohort, which confirmed favorable efficacy and acceptable safety in expert centers. In addition, Yamamoto et al. demonstrated that sporadic neoplasias detected in patients with UC have distinct characteristics and prognosis. Together, these reports highlight the importance of appropriate lesion characterization and support ESD as a valuable treatment option in carefully selected cases [26,27].

In patients diagnosed with UCAN, a non-negligible incidence of synchronous and metachronous multifocal carcinogenesis arising from chronically inflamed colonic mucosa has been reported, irrespective of the depth of wall invasion of the index lesion [1,2,3,4,5]. From this perspective, total proctocolectomy should be considered as a treatment option for UCAN to address the risk of occult or future neoplastic lesions throughout the colon. Conversely, when a comprehensive evaluation of the resected specimen, including architectural features, margin status, and background mucosal changes, supports a diagnosis of sporadic neoplasia rather than UCAN, avoiding total proctocolectomy may be feasible. In such cases, organ-preserving management can be considered, provided that patients undergo strict, long-term endoscopic surveillance at specialized centers [1,2,3,4,5].

Current surveillance strategies should be interpreted considering both general post-polypectomy guidance and IBD-specific recommendations. The British Society of Gastroenterology guideline provides a risk-stratified framework for post-polypectomy and post-colorectal cancer resection surveillance in the general population, aiming to optimize colonoscopic follow-up according to recurrence risk [28]. In contrast, the ECCO guideline emphasizes malignancy surveillance in patients with IBD, considering chronic inflammation, colitis extent, and cumulative cancer risk [29]. Together, these guidelines indicate that surveillance in IBD should not simply follow sporadic colorectal neoplasia algorithms but instead requires individualized assessment incorporating both lesion- and disease-specific factors.

### Limitations

This study had some limitations. First, this was a retrospective, single-center analysis with a limited number of EUS-evaluated cases. Thus, potential verification bias was likely present. Second, the examinations were not evaluated by multiple observers; all procedures were performed by a single experienced endoscopist. Therefore, larger multicenter studies are required to validate the study findings. Nevertheless, our findings emphasize that the differentiation between UCAN and sporadic neoplasia requires an integrated assessment that incorporates macroscopic morphology, surface structure, inflammatory background, and biopsy findings. Finally, all cases in this study were in endoscopic mucosal remission (up to MES 1). Clinical remission and endoscopic remission are prerequisites for the endoscopic diagnosis of UCAN. Recent developments, such as vascular pattern intensity analysis and artificial intelligence-based evaluation of pit architecture, are promising, and machine-learning-based diagnostic models tailored to UCAN may further enhance diagnostic accuracy in the future [30,31].

Overall, in this study, the use of ESD as a total biopsy enabled the comprehensive assessment of lesion extent, depth of invasion, and histologic differentiation. *En bloc* ESD provides both diagnostic and therapeutic benefits and represents a valuable strategy for addressing the uncertainty in preoperative diagnosis. Confirming shallow submucosal invasion using EUS and selecting ESD as a total biopsy may improve diagnostic precision and therapeutic outcomes in UCAN management.

## 5. Conclusions

An integrated approach that combines optical diagnosis, EUS-based depth assessment, and diagnostic ESD may enhance the accurate differentiation of UCAN from sporadic neoplasia. In particular, diagnostic ESD offers comprehensive histological evaluation of the entire lesion and may support clinical decision-making, including the potential avoidance of unnecessary total proctocolectomy in selected cases.

However, given the retrospective design and the limited number of cases, especially in the EUS analysis, these findings should be interpreted as exploratory. Further validation through larger, prospective multicenter studies is warranted to confirm the clinical utility of this stepwise strategy.

## Figures and Tables

**Table 1:** Number of lesions treated by each endoscopic procedure compared with the final histopathological diagnoses.

Endoscopic Therapy Pathological Results	EMR/CSP/ HSP	ESD		Total
		Suspected Sporadic Adenoma	Suspected UCAN	
Sporadic neoplasia	141	7	6	154
Hyperplastic polyp	28	1	0	29
Inflammatory polyp	20	0	1	21
UCAN	0	0	8	8
Total	189	8	15	212

Notes: CSP, cold snare polypectomy; EMR, endoscopic mucosal resection; ESD, endoscopic submucosal dissection; HSP, hot snare polypectomy; UCAN, ulcerative colitis-associated neoplasia.

**Table 2:** Clinicopathological characteristics and clinical outcomes of 23 lesions treated with ESD in UC-affected mucosa.

Case	Location	Tumor Size	Morphology		Pit Pattern Classification		JNET Classification		**Endoscopic Morphologic Pattern (Takabayashi Classification)**	**Endoscopic Diagnosis**
1	Rectum	12	0-IIc		IIIs		2B		Small round	UCAN
2	Sigmoid	12	0-IIa		IIIL, IV		2A		Gyrus-like	UCAN
3	Transverse	20	0-Isp		IV		2A		Gyrus-like	UCAN
4	Transverse	30	0-IIc		II		1		Gyrus-like	SSL
5	Ascending	40	0-IIa+Is		II		2A		Gyrus-like	TA
6	Sigmoid	10	0-IIa		IV, II, VI (mild)		2A		Gyrus-like	UCAN
7	Transverse	12	0-IIc		IIIs		2A		Small round	UCAN
8	Rectum	30	0-IIa		IV		2A		Gyrus-like	TA
9	Sigmoid	17	0-IIa+IIc		VI (mild)		2A		Unclassifiable	UCAN
10	Rectum	12	0-IIc		VI (mild)		2A		Small round	UCAN
11	Rectum	25	0-IIa+Is		IIIL, IV		2A		Small round	TA
12	Rectum	20	0-IIa		VI (mild)		2B		Gyrus-like	UCAN
13	Rectum	60	0-IIa		VI (mild)		2A		Gyrus-like	UCAN
14	Transverse	40	0-IIa		VI (mild)		2B		Mesh	UCAN
15	Transverse	15	0-IIa		II		1		Small round	SSL
16	Transverse	10	0-IIa+IIc		II, VI (mild)		2B		Small round	UCAN
17	Rectum	20	0-IIa		IV, VI (mild)		2B		Small round	UCAN
18	Sigmoid	20	0-IIa		IIIL		2A		Gyrus-like	TA
19	Ascending	15	0-IIa		II		1		Gyrus-like	SSL
20	Ascending	20	0-IIa		II		1		Gyrus-like	SSL
21	Rectum	50	0-IIa		IIIs, IV		2A		Mesh	UCAN
22	Rectum	30	0-IIa		IIIs, IV		2A		Mesh	UCAN
23	Transverse	20	0-IIa+Is		IV		2A		Gyrus-like	UCAN
EUS Depth of Tumor Invasion	Histological Type	E- Bloc Resection	Curative Resection	Adverse Events	Procedure Duration (min)	Duration of Hospitalization (Day)	Interval Between Diagnosis and ESD (Year)	Interval Between ESD and the Most Recent Colonoscopy (Year)	Metachronous Recurrence	Clinical Course
m	UCAN (cancer)	+	+	−	57	6	29	3	+	Total proctocolectomy
m	UCAN (HGD)	+	+	−	82	4	30	2	+	Total proctocolectomy
−	Inflammatory polyp	−	+	−	303	6	27	6	−	Follow-up endoscopy
−	SSL	+	+	−	54	3	48	7	−	Follow-up endoscopy
−	TA	+	+	−	110	13	1	4	−	Follow-up endoscopy
m	UCAN (HGD)	+	+	−	148	7	27	10	−	Total proctocolectomy
m	TA	+	+	−	16	6	28	9	−	Follow-up endoscopy
−	TA	+	+	−	68	7	6	−	−	Death from other causes
sm-d	UCAN (cancer)	+	−	−	72	6	17	8	−	Total proctocolectomy
−	TA	+	+	−	105	7	7	7	−	Follow-up endoscopy
−	TA	+	+	−	46	5	0	2	−	Follow-up endoscopy
−	TA	+	+	−	13	5	15	6	−	Follow-up endoscopy
−	TA	+	+	−	506	7	23	7	−	Follow-up endoscopy
−	TA	−	+	Delayed perforation	91	14	0	2	−	Follow-up endoscopy
−	SSL	+	+	−	14	6	2	1	−	Follow-up endoscopy
m	UCAN (LGD)	+	+	−	23	7	18	8	−	Follow-up endoscopy
m	UCAN (LGD)	+	+	−	275	5	19	7	−	Follow-up endoscopy
m	Carcinoma (sporadic)	+	+	−	66	6	0	−	−	Follow-up endoscopy
−	SSL	+	+	−	33	5	18	1	−	Follow-up endoscopy
−	SSL	+	+	−	36	5	18	1	−	Follow-up endoscopy
m	UCAN (LGD)	+	+	−	250	9	17	6	−	Follow-up endoscopy
m	UCAN (LGD)	+	+	−	200	9	17	4	−	Follow-up endoscopy
−	TA	+	+	−	120	8	25	−	−	Follow-up endoscopy

Note: This table summarizes the clinicopathological characteristics, endoscopic findings, pathological diagnoses, and clinical outcomes of 23 lesions treated with ESD in UC-affected mucosa. Tumor size is expressed in mm. EUS depth of tumor invasion is shown for lesions in which endoscopic ultrasonography was performed. “m” indicates an intramucosal lesion, and “sm-d” indicates a deeply invasive submucosal lesion. “+” indicates presence/achievement, and “−” indicates absence/non-achievement. ESD, endoscopic submucosal dissection; UC, ulcerative colitis; JNET, Japan Narrow-Band Imaging Expert Team; EUS, endoscopic ultrasonography; HGD, high-grade dysplasia; LGD, low-grade dysplasia; SSL, sessile serrated lesion; TA, tubular adenoma; UCAN, ulcerative colitis-associated neoplasia.

**Table 3:** Diagnostic performance of endoscopic ultrasonography for predicting T1b or deeper invasion.

① 2 × 2 Contingency Table					
	Pathology pT1b+		Pathology pT1a−		Total
EUS cT1b+	1 (TP)		0 (FP)		1
EUS cT1a−	1 (FN)		8 (TN)		9
Total	2		8		10
**② Diagnostic Performance**					
**Parameter**		**Estimate**		**95% CI (Clopper–Pearson)**	
Sensitivity		50.0% (1/2)		1.3–98.7%	
Specificity		100% (8/8)		63.1–100%	
PPV		100% (1/1)		2.5–100%	
NPV		88.9% (8/9)		51.8–99.7%	
Accuracy		90.0% (9/10)		55.5–99.7%	

Notes: Positive definition: pT1b or deeper. CI, confidence interval; EUS, endoscopic ultrasonography; NPV, negative predictive value; PPV, positive predictive value.

**Table 4:** Logistic regression analysis to compare ulcerative colitis-associated neoplasia (UCAN) with non-UCAN lesions.

Factor	Analysis			
	*p*-Value	OR	95% CI (Lower)	95% CI (Upper)
Age at treatment	0.226	1.050	0.970	1.135
Disease duration	0.127	0.935	0.858	1.019
Endoscopic Mayo score	0.509	1.647	0.374	7.245
Lesion size	0.809	1.008	0.944	1.076
Sex				
Male	–	1.000	–	–
Female	0.955	0.929	0.071	12.136
Disease extension				
Total	–	1.000	–	–
Left-sided, rectal	0.782	1.333	0.173	10.254
Location				
R and S	–	1.000	–	–
D, T, and A	0.187	3.333	0.557	19.949
Macroscopic appearance				
Type 0-IIa only	–	1.000	–	–
Others	0.673	0.686	0.119	3.963
Macroscopic appearance (elevation)				
Elevated type	–	1.000	–	–
Depressed type	0.196	4.667	0.451	48.257
Color				
Reddish	–	1.000	–	–
Pale/same color	0.049	6.667	0.987	45.036
NBI (JNET classification)				
1 ▪ 2A	–	1.000	–	–
2B	0.196	0.214	0.021	2.216
Pit pattern				
II ▪ III ▪ IV	–	1.000	–	–
VI	0.049	0.095	0.009	0.985
Takabayashi classification				
Small round ▪ mesh	–	1.000	–	–
Gyrus ▪ ripple	0.202	3.429	0.516	22.802

Notes: Analysis: Logistic regression. Significance level: 5% (α = 0.05). Software: SPSS (v.27). Outcome variable coding: UCAN = 1; non-UCAN = 0. The non-UCAN category includes sessile serrated lesions (SSLs), tubular adenomas (TAs), and inflammatory polyps. Additional note: Outcome categories were re-classified for regression analysis. CI, confidence interval; JNET, Japan Narrow-Band Imaging Expert Team; NBI, narrow-band imaging; OR, odds ratio.

## Data Availability

The original contributions presented in this study are included in the article. Further inquiries can be directed to the corresponding author.

## Abbreviations

The following abbreviations are used in this manuscript:

CSP	Cold snare polypectomy
EMR	Endoscopic mucosal resection
ESD	Endoscopic submucosal dissection
EUS	Endoscopic ultrasonography
HGD	High-grade dysplasia
HSP	Hot snare polypectomy
IBD	Inflammatory bowel disease
JNET	Japan Narrow-Band Imaging Expert Team
LGD	Low-grade dysplasia
MES	Mayo Endoscopic Subscore
NBI	Narrow-band imaging
NPV	Negative predictive value
PPV	Positive predictive value
SSL	Sessile serrated lesion
UC	Ulcerative colitis
UCAN	Ulcerative colitis-associated neoplasia
